# Uterine rupture during second-trimester abortion with misoprostol: a case report

**DOI:** 10.1093/jscr/rjaf233

**Published:** 2025-04-19

**Authors:** Suleiman A Belay, Michael A Negussie, Yirga W Alemu, Balemlay S Andualem, Eyasu F Yitina, Wondwosen M Dereje, Mikiyas G Teferi

**Affiliations:** School of Medicine, College of Medicine and Health Sciences, University of Gondar, Maraki Street, Gondar City, Central Gondar Zone, PO Box 196, Gondar, Ethiopia; School of Medicine, College of Health Sciences, Addis Ababa University, Tikur Anbessa Specialized Hospital, Churchill Avenue, Lideta Sub-City, PO Box 5657, Addis Ababa, Ethiopia; Department of Gynecology and Obstetrics, School of Medicine College of Medicine and Health Sciences, University of Gondar, Maraki Street, Gondar City, Central Gondar Zone, PO Box 196, Gondar, Ethiopia; Department of Gynecology and Obstetrics, School of Medicine College of Medicine and Health Sciences, University of Gondar, Maraki Street, Gondar City, Central Gondar Zone, PO Box 196, Gondar, Ethiopia; Department of Gynecology and Obstetrics, School of Medicine College of Medicine and Health Sciences, University of Gondar, Maraki Street, Gondar City, Central Gondar Zone, PO Box 196, Gondar, Ethiopia; School of Medicine, College of Medicine and Health Sciences, University of Gondar, Maraki Street, Gondar City, Central Gondar Zone, PO Box 196, Gondar, Ethiopia; School of Medicine, College of Health Sciences, Addis Ababa University, Tikur Anbessa Specialized Hospital, Churchill Avenue, Lideta Sub-City, PO Box 5657, Addis Ababa, Ethiopia

**Keywords:** misoprostol, uterine rupture, second-trimester abortion, unscarred uterus, case report

## Abstract

Misoprostol, a prostaglandin E1 analog, is widely used in obstetrics for abortion induction and labor management. Although generally safe, it can lead to rare complications such as uterine rupture, even in patients without prior uterine scarring. This case report describes a 27-year-old gravida 3, para 1 woman with no history of uterine surgery who presented with a second-trimester missed septic abortion. After receiving six sublingual doses of misoprostol (400 μg each) without fetal expulsion, she developed signs of sepsis, including fever, tachycardia, and leukocytosis. Ultrasound revealed an empty uterus with a defect in the anterior wall and the fetus and placenta in the peritoneal cavity. Emergency laparotomy confirmed a complete transverse rupture of the lower uterine segment with necrotic edges. The fetus and placenta were extracted, the uterus was repaired, and the patient recovered well with postoperative antibiotics, being discharged after 7 days. This case highlights uterine rupture as a rare but serious complication of second-trimester abortion with misoprostol, emphasizing the importance of careful monitoring and prompt recognition of complications to ensure patient safety.

## Introduction

Misoprostol, a prostaglandin E1 analog, is approved by the Food and Drug Administration for preventing gastric ulcers caused by long-term treatment with nonsteroidal anti-inflammatory drugs [1]. Due to its uterotonic effect, it has found increasing use in obstetrics for purposes such as abortion induction, cervical ripening, labor induction, and treatment of postpartum hemorrhage due to uterine atony [2–4]. Its popularity, particularly in the developing world, is attributed to its effectiveness, low cost, and stability at room temperature [4]. Uterine rupture is a serious and often life-threatening condition for both mothers and fetuses, occurring in ~1% of pregnancies involving a previously scarred uterus during labor induction with misoprostol or oxytocin [5]. Isolated cases of unscarred uterine rupture following misoprostol use for cervical ripening have also been reported [6, 7], as well as uterine rupture during second-trimester abortions induced with misoprostol [8–10]. We report our experience with a patient who received sublingual misoprostol as an abortifacient in the second trimester of pregnancy and subsequently experienced a uterine rupture.

## Case presentation

A 27-year-old gravida 3, para 1 woman with a history of one complete spontaneous abortion and no prior cesarean sections or other uterine surgeries, was referred from a local health center with a diagnosis of second-trimester missed septic abortion. Her gestational age was 23 weeks and 2 days based on her last normal menstrual period. She had presented with complaints of absent fetal movement for 1 day, accompanied by foul-smelling vaginal discharge and low-grade fever with chills and rigor for 3 days. She was administered six doses of sublingual misoprostol (400 μg each) every 4 h for 24 h but failed to expel. She was also started on ceftriaxone and metronidazole.

On examination, her blood pressure was 100/60 mm Hg, with a tachycardia of 120 beats per minute, a respiratory rate of 22 breaths per minute, and a temperature of 38.0°C. There was a 24-week gravid uterus with no fetal heartbeat and diffuse abdominal tenderness but no there was no signs of uterine hyperstimulation. Pelvic examination revealed a cervix that was 2 cm dilated and 60% effaced, with a high fetal station.

Laboratory investigations showed leukocytosis (16.53 × 10^3^/μl) with 90.3% neutrophils. Her creatinine level was elevated at 1.22 mg/dl, while other blood parameters were within normal ranges. Obstetric ultrasound revealed an empty uterus with a visible slit and a defect in the anterior wall. The placenta and fetus were located in the peritoneal cavity with minimal fluid collection.

**Figure 1:**
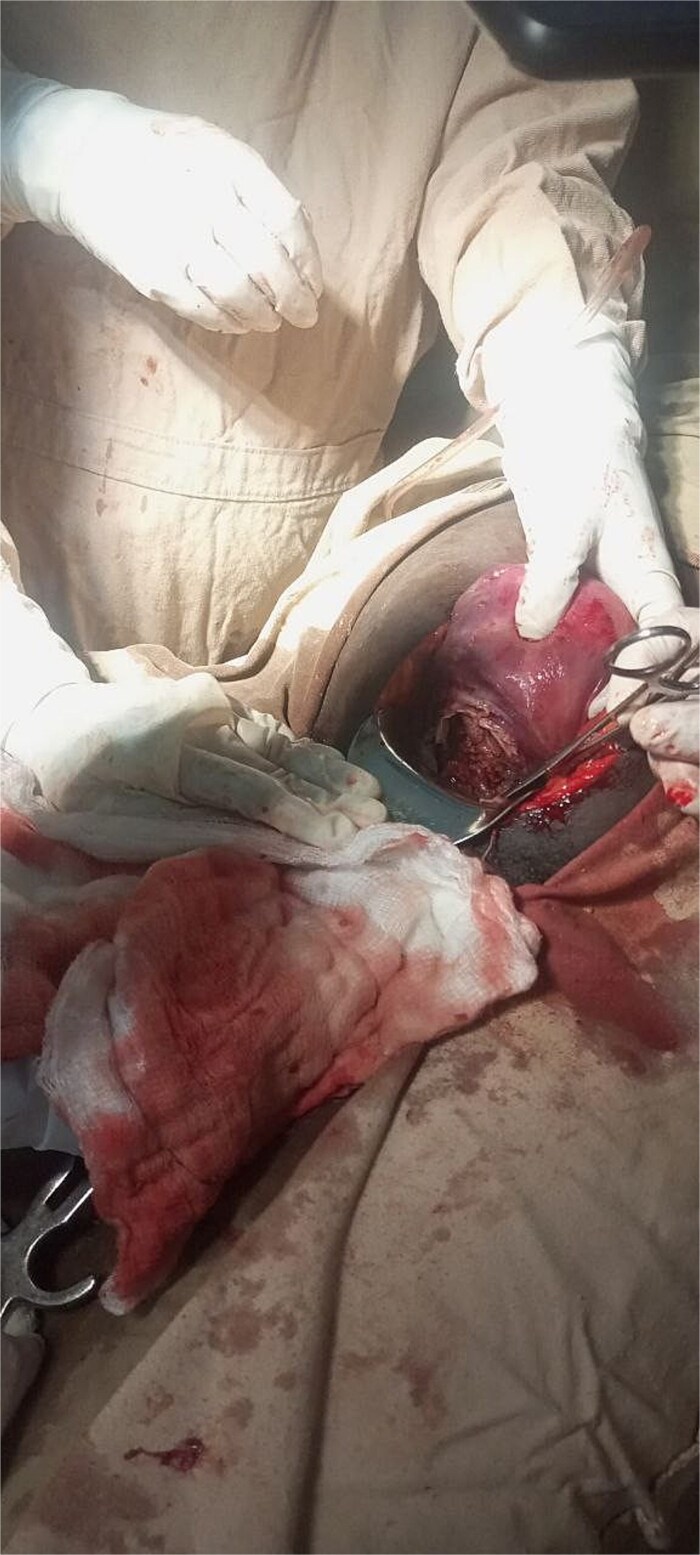
Intraoperative image taken showing complete transverse rupture over the lower uterine segment with necrotic edge.

With a provisional diagnosis of second-trimester missed septic abortion and possible uterine rupture, the patient underwent a laparotomy. Intraoperatively, upon entry, there was a gush of air and foul odor. A grade 3 macerated fetus and placental tissue were found inside the peritoneal cavity. There was a complete transverse rupture of the lower uterine segment with necrotic edges (Fig. 1). Approximately 100 ml of thin pus mixed with reactive fluid and edematous bowel was present.

There was no major vessel involvement, and the bilateral tubes and ovaries appeared normal. The dead fetus and placental tissue were extracted from the peritoneal cavity, and the edges of the ruptured site were trimmed. The uterus was repaired with two layers of vicryl number 1 sutures. The abdomen was thoroughly washed with warm saline.

She experienced a smooth postoperative course and received a 5-day regimen of broad-spectrum antibiotics, including ampicillin, ceftriaxone, and metronidazole. Her creatinine levels were normalized, and her post op hematocrit was normal too. The patient was discharged after a 7-day hospital stay. During follow-up, she was doing well with healing wounds.

## Discussion

Early pregnancy failure is common, occurring in 15%–20% of known pregnancies [11]. Termination of early pregnancy failure can be achieved through expectant management, surgical evacuation, or medical management [12]. Misoprostol alone for termination of pregnancy (TOP) was first described in 1994 and has since been widely used for TOP in the normal uterus [13, 14]. Misoprostol can be administered vaginally, orally, or in combination for first and second-trimester TOP [15]. The dose of misoprostol used for labor induction varies across studies, ranging from 25 to 100 mg, with a maximum dose of 600 mg reported in some studies [19]. Our patient received six sublingual doses totaling 2400 μg, which exceeds most recommendations.

Rupture of an intact uterus is a rare but serious obstetric emergency that can occur at any time during pregnancy or labor, either spontaneous or induced. The incidence of this catastrophic event is reported to be 0.03%–0.08% among all pregnant women [16]. Uterine rupture with the use of misoprostol is more frequently reported in multiparous women and those with uterine scars, typically occurring at term rather than in the second trimester [17]. Among women with a prior cesarean delivery undergoing second trimester abortion using misoprostol, the risk of uterine rupture is less than 0.3% [20]. The American College of Obstetricians and Gynecologists recommends avoiding misoprostol use in women with a prior cesarean birth due to the risk of uterine rupture [18]. Our patient was a multiparous woman with no previous uterine scars or surgical procedures.

Although the maternal risk of mortality is low, life-threatening conditions such as hemorrhagic shock and other complications may arise following a uterine rupture. In our case, the patient presented early, there was no significant bleeding, and uterine repair was successfully performed.

## Conclusion

Misoprostol is generally safe for second-trimester termination of pregnancy; however, rare complications such as uterine rupture can never be wholly prevented. The drug should be used with caution and under close observation.
